# The ups and downs of paclitaxel-coated balloons and paclitaxel eluting stents: do the conclusions of SWEDEPAD 2 change our practice? Should we be concerned about mortality (again)?

**DOI:** 10.1186/s42155-025-00645-9

**Published:** 2026-01-27

**Authors:** Stefan Müller-Hülsbeck, Fabrizio Fanelli, Patrick Haage, Mohamad Hamady, Hicham Kobeiter, Romaric Loffroy, Gerard O’Sullivan, Lakshmi A. Ratnam, Catharina S. P. van Rijswijk, Maria Antonella Ruffino, Florian Wolf, Robert A. Morgan

**Affiliations:** 1 DIAKO Hospital gGmbH, Flensburg, Kiel, Germany; 2 Vascular and Interventional Radiology Department, “Careggi” University Hospital–University of Florence, Florence, Italy; 3 Helios University Hospital, University Witten/Herdecke, Wuppertal, Germany; 4 Imperial College, St. Mary’s Campus, London, UK; 5 Radiology Department, H. Mondor Hospital, Assistance Publique-Hôpitaux de Paris, University Paris Est Creteil, Creteil, France; 6 Department of Diagnostic and Interventional Radiology, François-Mitterrand University Hospital, Dijon, France; 7 University Hospital Galway, Galway, Ireland; 8 George’s Healthcare NHS Trust, St. George’s University Hospitals NHS Foundation Trust, London, UK; 9 School of Health & Medical Sciences, City St. George’s, University of London, London, UK; 10 Department of Radiology, Leiden University Medical Center, C2-S Leiden, The Netherlands; 11 Interventional Radiology, Institute of Imaging of Southern Switzerland-EOC Lugano, Lugano, Switzerland; 12 Division of Cardiovascular and Interventional Radiology, Medical University of Vienna, Vienna, Austria; 13 St. George’s University of London, London, UK

## Abstract

The use of paclitaxel-coated devices (PCDs) in peripheral arterial disease continues to generate interest and controversy. The PCD journey to date has been a story of highs and lows. The recently published SWEDEPAD 1 and 2 trials have rekindled discussion regarding efficacy and a potential link to mortality.

## Up

The efficacy of Paclitaxel DCB (drug-coated balloons) and DES (drug-eluting stents) has been proven in several well-regarded landmark trials [1–3] DCBs achieved up to 82.2% primary patency and 2.4% freedom from TLR (target lesion revascularization) at 1 year, sustained over 3 years with 69.5% and 15.2% [4] compared to PTA alone (52.4% and 20.6% at 1 year; 45.1% and 31.1% at 3 years). The efficacy of DES has been proven for two DES—the ZilverPTX and the Eluvia DES. For the ZilverPTX trial, 5 years of data are available with primary patency and freedom from TLR of 66.4% and 83.1%, respectively, compared to optimized treatment with PTA and/or bare metal stents reaching 43.4% and 67.6% [5]. Data for the Eluvia DES are available in the MAJESTIC trial and the IMPERIAL trial indicating primary patency and freedom from TLR at 2 years in 83% and 87.3%, respectively [6–9]. These encouraging data were responsible for an increasingly widespread use of drug-based therapies in PAD treatment in femoropopliteal disease up to 2018.

### Down

In 2018, Katsanos et al. published their meta-analysis of randomized controlled trial data using drug-based therapies with paclitaxel coated DCBs and DES in claudicants in the femoropopliteal segment. They reported a mortality signal indicating increased death from all causes of 14.7% at 5 years using Paclitaxel, whereas all cause death after PTA was 8.1% at 5 years [10].

Following publication of the Katsanos meta-analysis, endovascular practitioners and industry leaders immediately initiated major efforts to review the datasets that were used by Katsanos et al. New meta-analyses from similar patient pools (existing and new patients included) that included subsequent assessment for mortality were conducted and published [11–16]. These and other studies on randomized industry patient-level data, independent randomized data and observational data that also assessed specifically for a link to mortality as well as patency and TLR were initiated. The outcomes from these analyses that are all now in the public domain repudiated Katsanos’s original conclusions [17–19] and moreover convinced the FDA and the vascular community at large that no mortality risk should be ascribed to the use of paclitaxel-coated devices in infrainguinal revascularization procedures.

It is worth mentioning for inclusivity that despite Circulation’s formal correction of the ZilverPTX data in February 2019, which acknowledged a mistake regarding the reversed mortality figures showing the 5-year all-cause mortality rate was 13.6% (16.9% for the primary DES group and 10.2% for the PTA group, *P* = 0.03), *no deaths were judged to be procedure or device related* [5]. Regarding the In.Pact paclitaxel DCB, in a March 2019 statement, Medtronic noted a programming error in the clinical data reporting, isolated to the 2- and 3-year follow-up periods in the postmarket study. The error affected the In.Pact paclitaxel safety analysis published in JACC [20]. According to Medtronic, the initial conclusions from the patient level meta-analysis remain intact, i.e., at 5 years, there was no statistically significant difference in all-cause mortality between the DCB and plain percutaneous transluminal angioplasty (PTA) arms. It should be restated that the above statements from Circulation and Medtronic were several years ago, and until Swedepad, no studies that included mortality as an outcome (primary or secondary) have found any relationship between paclitaxel devices and mortality.

## Up

A robust body of evidence now exists to negate the existence of a long-term mortality signal associated with PCDs, i.e., any mortality risk is negligible or absent [11–19]. Finally, all efforts to identify a link and a causal explanation for the perceived mortality risk between Paclitaxel dose and mortality have failed. None of the studies have found a single cause or group of similar causes that might explain mortality following PCDs [19, 21]. These findings resulted in an increased use of Paclitaxel drug-based devices again based on their published efficacy and FDA’s announcement on July 11, 2023, to health care providers on “Paclitaxel-Coated Devices to Treat Peripheral Arterial Disease Unlikely to Increase Risk of Mortality” (https://www.fda.gov/medical-devices/letters-health-care-providers/update-paclitaxel-coated-devices-treat-peripheral-arterial-disease-unlikely-increase-risk-mortality#:~:text=None%20of%20these%20studies%2C%20with,Previous%20FDA%20Communications).

## Down again?

The Swedish Drug-Elution Trial in Peripheral Arterial Disease 2 (SWEDEPAD 2), multicenter, participant-masked, registry-based, randomized controlled evaluation conducted at 22 Swedish vascular centers included patients suffering from intermittent claudication (Rutherford categories 1–3) between November 2014 and September 2023. The primary efficacy endpoint was the between-group difference in quality of life at 1 year, assessed with the 6-item Vascular Quality of Life Questionnaire (VascuQoL-6), a peripheral artery disease-specific quality of life instrument. Femoropopliteal interventions were performed in 1092 patients (96·1%). At 1 year, VascuQoL-6 scores did not differ between groups (mean difference − 0·02 [95% CI − 0·66 to 0·62]; *p* = 0·96).

The authors concluded that patients with Rutherford stage 1–3 peripheral artery disease undergoing infrainguinal endovascular revascularization, paclitaxel-coated devices did not improve disease-specific quality of life at 1 year compared with uncoated devices when using paclitaxel-coated devices.

Although not the primary objective of the trial, regarding mortality, all-cause mortality did not differ over a median 7.1 years (IQR 3.9–8.2); hazard ratio (HR) 1.18 (95% CI 0.94–1.48); *p* = 0.16. There was no difference in mortality up to 4 years, but the mortality incidence at 5 years was higher in patients randomly assigned to the paclitaxel-coated devices group (4.57 vs 3.28 per 100 person-years; HR 1.47 [95% CI 1.09–1.98]; *p* = 0·010) [22]. This increased mortality at 5 years disappeared after 5 years, as far out as 10 years follow-up.

The clinical outcomes of SWEDEPAD 2 (equivalence to non-PCDs) raise the question again, should we reduce or stop using paclitaxel-coated devices for the treatment of lifestyle-limiting claudication in patients with Rutherford stage 1–3?

SWEDEPAD 2 is a major randomized controlled trial, and RCTs remain the cornerstone of evidence-based medicine for causal inference.

However, several aspects of the SWEDEPAD 2 trial design and interpretation warrant caution when drawing broad conclusions regarding the clinical value and safety of paclitaxel-coated devices.

First, the choice of a single patient-reported outcome (VascuQoL-6 at 12 months) as the primary efficacy endpoint may be insufficiently sensitive to detect differences driven by restenosis and target–vessel reintervention. In patients with intermittent claudication, early patency is often preserved across treatment strategies at 1 year, potentially masking device-related benefits that may be clinically relevant but not reflected in short-term quality-of-life scores.

Second, the study pooled heterogeneous drug-coated balloons and stents with differing paclitaxel doses and coating technologies. This pragmatic design assumes a class effect and may dilute device- or dose-specific efficacy and safety signals. Device-level analyses would be essential before concluding the absence of benefit or the presence of harm across the entire drug-coated device class.

Third, the interpretation of long-term mortality findings deserves particular caution. SWEDEPAD-2 was not primarily powered for all-cause mortality, and subgroup- or time-window-specific mortality signals are vulnerable to chance findings and residual confounding. Such observations should therefore be considered hypothesis-generating and interpreted in the context of the broader and still mixed evidence base, including randomized trials, registries, and patient-level meta-analyses.

Finally, the exclusive focus on intermittent claudication limits generalizability. In this population, symptom improvement from exercise therapy and optimal medical management may overshadow device-related effects, whereas outcomes and risk–benefit profiles may differ substantially.

## No-keep going up

A French nationwide analysis of > 250,000 patients undergoing lower extremity revascularization between October 2011 and December 2019 found reduced mortality associated with paclitaxel-coated device use. The study included 259,137 patients, of whom 20,083 (7.7%) were treated with ≥ 1 paclitaxel-coated device. After a median follow-up of 4.1 years (interquartile range, 2.3–6.4 years), the rate of death was 7.3/100 patient-years in the paclitaxel-coated device group and 10.4/100 patient-years in the control group. After adjusting for confounding factors, paclitaxel-coated device treatment was associated with a lower risk of mortality (hazard ratio, 0.86; 95% confidence interval, 0.84–0.89) [23]. The large size and robust set of statistical methods used in this analysis should provide reassurance to patients, clinicians, and policymakers that paclitaxel-coated devices are safe for use in lower extremity endovascular revascularization. In another large database, an assessment of real-life data from 64,771 patients in Germany who received 200,681 devices found no evidence for increased mortality associated with paclitaxel-based drug-eluting devices over 11 years of follow-up [17]*.*

Returning to SWEDEPAD 2, the triallists used quality of life as the primary end-point rather than primary patency and TLR, which have been used in most previous trials. Moreover, the SWEDEPAD 2 trial used generic “drug-coated devices” which were a mix of drug-coated balloons (DCBs) with varying efficacy and drug-eluting stents (DES) that delivered paclitaxel, rather than specific brand-name devices. The study did not specify particular models but focused on the paclitaxel-coating itself as the intervention for patients with intermittent claudication. These generic “drug-coated devices” have also been used in huge patient cohorts as mentioned above in France and Germany, again, where no evidence for increased mortality associated with paclitaxel-based drug-eluting devices was found for over 11 years [17].

Comparing datasets with different primary endpoints like primary patency and target lesion revascularization or quality of life and drawing conclusions from the comparison is challenging. Arguably, the more questionable conclusions are from the SWEDEPAD 2 trial, which did not find evidence to support the routine use of paclitaxel-coated devices for patients with intermittent claudication, questioning their overall clinical benefit and raising concerns about their risk–benefit profile.

Finally, but importantly, there are diverging findings of randomized and real-world studies, which have led some authors to state that most real-world data patients would never be eligible to be assigned to RCTs due to their strict inclusion and exclusion regime [24]. So do RCT outcomes really reflect outcomes in the real world?

What about SWEDEPAD 1 [25] and CLTI patients? CLTI patients are a very different patient population from claudicants, and primary endpoints in CLTI trials generally include amputation-free survival and wound healing rather than patency and TLR. In SWEDEPAD 1, the primary efficacy endpoint was ipsilateral major amputation (above the ankle) during follow-up. Two thousand three hundred fifty-five patients were included in the intention-to-treat analysis (1180 in the paclitaxel-coated group and 1175 in the uncoated group). There was no significant difference in the rate of ipsilateral major amputation between using paclitaxel-coated or uncoated devices (hazard ratio [HR] 1.05 [95% CI 0.87–1.27]; *p* = 0.61) with a maximum of 5 years of follow-up. There was no difference in all-cause mortality (HR 1.04 [95% CI 0.92–1.17]; *p* = 0.54). Based on these findings, the authors could conclude that for patients with chronic limb-threatening ischemia undergoing infrainguinal endovascular revascularization, paclitaxel-coated devices did not reduce major ipsilateral amputations compared to non-coated devices. Multi-level disease patterns, vessel calcification, and a different composition of severe coexisting morbidities may also contribute to this finding. However, many vascular specialists remain committed to the principle that endovascular interventions using paclitaxel-based drug technology should be considered in patients with CLTI and FP occlusive disease [26].

While SWEDEPAD-2 provides valuable real-world data, its results should not be interpreted as definitive evidence against the efficacy or safety of paclitaxel-coated devices. Further device-specific analyses and pooled patient-level evaluations are needed to clarify both clinical benefit and long-term safety.

In our opinion, SWEDEPAD 2 does not change the general agreement among the majority of vascular specialists that paclitaxel-coated devices are safe to use, and are beneficial in terms of efficacy (increased primary patency and reduced TLR), in the femoropopliteal segment in patients with intermittent claudication (Rutherford categories 1–3).

Published on behalf of the Endovascular Subcommittee of the Cardiovascular and Interventional Radiology Society of Europe (CIRSE)

## Data Availability

Not applicable.
